# Headaches in juvenile systemic lupus erythematosus patients: a cross-sectional study

**DOI:** 10.1055/s-0045-1810407

**Published:** 2025-08-04

**Authors:** Bryan Silva Marques Cajado, Renata Lopes Francisco Andrade, Maria Angelina Carvalho Pereira, Marcelo Melo Aragão, Maria Teresa Terreri

**Affiliations:** 1 Universidade Federal de São Paulo, Escola Paulista de Medicina, Departamento de Neurologia e Neurocirurgia, São Paulo SP, Brazil.; 2 Universidade Federal de São Paulo, Escola Paulista de Medicina, Departamento de Pediatria, São Paulo SP, Brazil.

**Keywords:** Headache, Lupus Erythematosus, Systemic, Migraine Disorders, Quality of Life, Pediatrics

## Abstract

**Background:**

Juvenile systemic lupus erythematosus (jSLE) often involves the central nervous system, with headache being the most common symptom.

**Objective:**

To describe the frequency, characteristics, and impact on quality of life of headaches in jSLE patients.

**Methods:**

We conducted a cross-sectional study with jSLE patients under the age of 19 years through chart reviews and questionnaires. The participants underwent clinical and neurological exams, assessments of disease activity and damage, and evaluations of headache characteristics. Quality-of-life impacts were measured using the Brazilian Portuguese version of the Pediatric Quality of Life Inventory (PedsQL), and cognitive function was assessed with the Mini-Mental State Examination.

**Results:**

Out of 34 patients enrolled, 17 presented with headaches (which were more prevalent in female subjects). The age of the patients at the time of the evaluation ranged from 8 to 18 years, and the mean age at headache onset was of 11.3 years. Most participants with headaches reported having episodic headaches (76.5%), no perimenstrual crises (73.3%), and a family history of migraines (88.2%). Additionally, most patients reported physical activity (35.3%) and stress (58.8%) as triggers. Patients with headaches had significantly lower PedsQL scores compared with those without headaches, both in health and activities (61.76 versus 73.71 respectively; *p* = 0.04) and feelings (42.65 versus 60.35 respectively; *p* = 0.049).

**Conclusion:**

Headaches were more prevalent in female jSLE patients, and the most subjects experienced migraines as the primary cause of headache. Patients with headaches presented lower scores on quality-of-life questionnaires. These findings highlight the need for a comprehensive approach to headache management in jSLE to enhance patient well-being.

## INTRODUCTION

Systemic lupus erythematosus (SLE) is a chronic autoimmune disease mediated by autoantibodies that affects multiple organs and systems. 1 Juvenile SLE (jSLE) presents with more severe clinical manifestations, faster progression, and greater disease-associated damage than the adult presentation. 1 2 3 There is also a higher frequency of central nervous system (CNS) involvement compared with adult-onset SLE. 3 4

Neuropsychiatric (NP) manifestations in jSLE occur in between 20 and 40% of the patients, 3 5 6 and can be the initial manifestation of the disease in up to 65% of the cases. 6 The auxiliary criteria for the diagnosis of the disease, described by the American College of Rheumatology (ACR) in 1997, 7 consider only the presence of psychosis and/or epileptic seizures as NP manifestations compatible with SLE. In 2012, these criteria were updated by an international task force 8 (Systemic Lupus International Collaborating Clinics, SLICC) to increase the sensitivity of the manifestations that would be considered compatible with the disease. This update included the presence of epileptic seizures, psychosis, mononeuritis multiplex, myelitis, peripheral or cranial nerve neuropathy, and acute confusional state, as CNS involvement compatible with SLE. 4 7 8

Headache is a noteworthy complaint, as it is considered the most frequent symptom in both adult and pediatric populations. 3 9 There is still considerable difficulty in associating NP manifestations with disease activity, as they can be non-specific, and no biomarkers have been identified yet. 6

The present study aims to describe the frequency of headaches in children and adolescents with jSLE, as well as their main characteristics, and the impact on the patients' quality of life.

## METHODS

We conducted a cross-sectional study through chart review, interviews, and the application of questionnaires. Data was collected from a convenience sample of patients under the age of 19 years diagnosed with jSLE, according to the criteria proposed by the SLICC in 2012, 8 who were undergoing regular follow-up care at a tertiary Pediatric Rheumatology Service. Patients with other associated chronic diseases, those experiencing acute infections, or those who had undergone pulse therapy with methylprednisolone in the past month were excluded.

The patients underwent comprehensive clinical and neurological examinations performed by a pediatric neurologist. In addition, assessment of disease activity using the Systemic Lupus Erythematosus Disease Activity Index (SLEDAI) was performed. 10 Disease activity was considered present when the SLEDAI score was > 4. The SLICC criteria are essential to diagnose neuropsychiatric events in SLE. These criteria classify neuropsychiatric manifestations into categories such as seizures, psychosis, mood disorders, cognitive dysfunction, and peripheral neuropathy, each with specific diagnostic definitions. New-onset seizures must be directly linked to SLE and not attributable to other conditions. 8 The patients were also assessed regarding headache characteristics, as well as their perceptions and those of their parents of the impact of headache on overall quality of life using the Brazilian Portuguese version of the Pediatric Quality of Life Inventory (PedsQL), 11 and a cognitive assessment using the Mini-Mental State Examination, adapted for the pediatric age group. 12 The PedsQL scale scores from 0 to 92, with lower scores indicating better quality of life. The Mini-Mental State Examination scores from 0 to 37, with scores considered normal if greater than 25 for children aged 3 to 5 years, greater than 28 for children aged 6 to 8 years, greater than 30 for children aged 9 to 11 years, and greater than 35 for children aged 12 years or older. 12

The study included 34 patients, 17 with and 17 without headaches. In the descriptive analysis of the categorical variables of interest, absolute and relative frequencies were used, while for numerical variables, measurements of central tendency (such as mean and median), variation (such as minimum and maximum), and dispersion (such as standard deviation) were employed. To compare the categorical variables between the groups with and without headaches, the Chi-squared and the Fisher's Exact tests were applied. For the numerical variables, the Mann-Whitney test was used to compare the means or medians between the groups.

The study was approved by the Ethics in Reasearch Committee of Universidade Federal de São Paulo, under number 01446612.5.0000.5505. Informed consent forms (for the parents) and assent forms (for the underage patients) were provided and signed.

## RESULTS

There were 17 patients with headache. The age of the patients at the time of the evaluation ranged from 8 to 18 years, with a mean of 15.2 years among patients with headaches, and 14.8 years among those without headaches. The presence of headaches was more common in female subjects (85%). No differences between the groups regarding age and sex were observed.

Table 1 shows the clinical characteristics of the patients with headaches. The mean age at headache onset was of 11.3 years. Most participants with headaches reported having episodic headaches (76.5%), no perimenstrual crises (73.3%), and a family history of migraines (88.2%). Additionally, most patients reported physical activity (35.3%) and stress (58.8%) as triggers.

**Table 1:** Clinical characteristics of the patients with headaches ( *N* = 17)

Characteristics	N	%
Chronic headache Episodic headache	4	23.5
	13	76.5
Perimenstrual crisis	4*	26.7
Morning headache	6	35.3
Evening headache	3	17.7
Nocturnal headache	10	58.8
Frontal location	11	64.7
Parietal location	5	29.4
Temporal location	2	11.8
Occipital location	2	11.8
Holocranial location	1	5.9
Unilateral	11	64.7
Pulsatile pain	13	76.5
Heavy or squeezing pain	6	35.3
Nausea	3	17,7
Vomiting	1	5.9
Photophobia	12	70.6
Phonophobia	10	58.8
Olfactory sensitivity	2	11.8
Worsening with physical activity	10	58.8
Drowsiness during crisis	2	11.8
Worsening pain during jSLE activity	5	29.4
Pain improvement after pulse therapy	5**	35.7
Aura	4	23.5
Triggered by fasting	2	11.8
Triggered by food	1	5.9
Triggered by physical activity	6	35.3
Triggered by menstruation	3*	20.0
Triggered by stress	10	58.8
Triggered by conflict	7	41.2
Triggered by worry	3	17.7
Triggered by environmental factors	3	17.7
Triggered by odors	1	5.9
Recurrent abdominal pain	2	11.8
Familial history of migraine	15	88.2
Emergency room visit due to pain	6	35.3
Use of prophylactic medication	2	11.8
Use of abortive medication for attacks	17	100.0
Migraine	14	82.4
Tension headache	3	17.7

Abbreviation: jSLE, juvenile systemic lupus erythematosus.

Notes: *This data was not available for 2 patients; **this data was not available for 3 patients.

Table 2 shows the demographic and clinical data according to the presence or absence of headaches. None of the evaluated parameters showed a difference between the groups. Neuropsychiatric manifestations occurred in 17 patients (50%), with headaches being the most common (50%). Headaches were not associated with any other NP manifestation ( Table 3 ).

**Table 2:** Demographic and clinical data of patients according to the presence or absence of headache

Variable		Mean	SD	*p* -value
Current age (years)	With headache	14.8	± 2.3	0.553
	Without headache	15.2	± 2.7	
Age at diagnosis (years)	With headache	11.7	± 31	0.702
	Without headache	11.4	± 3.0	
Number of SLICC criteria	With headache	8.2	± 2.5	0.725
	Without headache	8.4	± 2.1	
SLEDAI score	With headache	3.2	± 3.1	0.335
	Without headache	4.8	± 4.8	

Abbreviations: SD, standard deviation; SLICC, Systemic Lupus International Collaborating Clinics; SLEDAI, Systemic Lupus Erythematosus Disease Activity Index.

**Table 3:** Neuropsychiatric manifestations of patients according to the presence or absence of headache

Neuropsychiatric manifestations	N (with headache)	N (without headache)	*p* -value
Neuropsychiatric symptoms	9	8	0.30
Seizure	4	3	0.67
Cerebrovascular disease	4	1	0.14
Mood disorder	3	1	0.28
Anxiety disorder	3	0	0.06
Psychosis	3	0	0.06
Aseptic meningitis	0	1	0.37
Demyelinating syndrome	1	0	0.31
Movement disorder	1	0	0.37
Cognitive impairment	1	0	0.37
Myasthenia gravis	1	0	0.37
Cranial neuropathy	1	0	0.37

Table 4 shows the mean scores of the study sample on the PedsQL and Mini-Mental State Examination questionnaires, which varied across different domains such as health and activities, feelings, interactions with others, school, physical capacity, emotional aspect, social aspect, and school functioning. Regarding the Mini-Mental State Examination, the overall mean score was of 34.41, with no difference between the groups.

**Table 4:** Scores of the patients on the PedsQL and Mini-Mental State Examination according to the presence or absence of headache

Variable		Median	SD	*p* -value
PedsQL: Patient – About my health and activities	With headache	61.76	± 13.91	0.040
	Without headache	73.71	± 16.80	
PedsQL: Patient – About my feelings	With headache	42.65	± 25.75	0.049
	Without headache	60.35	± 20.35	
PedsQL - Patient – About how I live with other people	With headache	70.88	± 15.54	0.122
	Without headache	78.24	± 27.38	
PedsQL: Patient – About school	With headache	57.06	± 19.85	0.135
	Without headache	67.06	± 19.61	
PedsQL: Caregiver – Physical capacity	With headache	64.26	± 21.53	0.342
	Without headache	69.58	± 25.75	
PedsQL: Caregiver – Emotional aspects	With headache	47.19	± 23.45	0.219
	Without headache	58.33	± 21.27	
PedsQL: Caregiver – Social aspects	With headache	70.94	± 22.08	0.379
	Without headache	77.33	± 23.82	
PedsQL: Caregiver – School activity	With headache	56.88	± 25.68	0.131
	Without headache	71.00	± 20.46	
Score on the Mini-Mental State Examination	With headache	34.06	± 2.30	0.462
	Without headache	34.76	± 1.56	

Abbreviations: PedsQL, Pediatric Quality of Life Inventory; SD, standard deviation.

**Figure 1:**
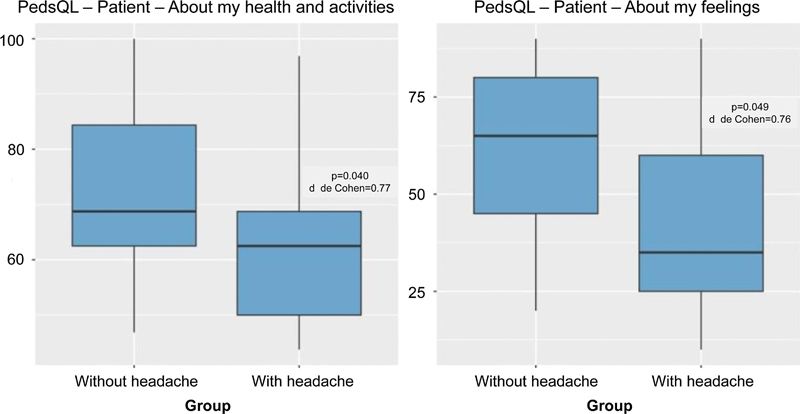
Scores of the study sample on the domains of the Pediatric Quality of Life Inventory (PedsQL) according to the presence or absence of headache.

The comparative analysis of the patients with and without headaches revealed significant differences in the PedsQL scores related to the patient1s perception of their health and activities, as well as their feelings. Concerning the PedsQL domain related to health and activities, patients with headaches had significantly lower scores compared with those without headaches (61.76 versus 73.71 respectively; *p* = 0.04), indicating that the presence of headaches is associated with a negative impact on the health and activities of the patients. Similarly, regarding feelings, patients with headaches had significantly lower scores on the PedsQL compared with those without headaches (42.65 versus 60.35 respectively; *p* = 0.049), suggesting that the presence of headaches is associated with a negative impact on the patients' feelings ( Figure 1 ).

## DISCUSSION

Headaches are a common complaint in patients with jSLE, and they are considered the most frequently observed NP symptom. 13 In the current study, we found that headaches occurred more frequently in female subjects. Patient age, disease damage, and disease activity indices were not associated with the presence of headaches.

Although lupus-related headaches exist, most lupus patients in the present study experienced migraines as the primary cause of this symptom. Symptoms such as phonophobia, photophobia, nausea and/or vomiting, pulsatile and unilateral pain, presence of aura, frontal predominance, nocturnal occurrence, association with emotional factors and menstruation, and family history, are some characteristics suggesting this diagnosis. Other authors 14 have also identified migraines as the primary cause of headaches in SLE.

Episodic headaches were observed in three-quarters of patients with headaches and in about one-third of jSLE patients, a frequency similar to that found in previous Brazilian records. 15

The diagnosis of migraine can be established through history-taking, physical examination, and specific questionnaires, thus avoiding the need for imaging in these cases. However, not all headaches are primary, and recent-onset or progressively-worsening headaches may be associated with infections, arterial hypertension, thrombotic events, or increased intracranial pressure, and they require imaging. 14 Magnetic resonance imaging is essential for the differential diagnosis; however, there are no specific radiological findings for NP SLE, and even in cases of CNS vasculitis, imaging may appear normal. 13 As no headaches with such characteristics were observed in the present study, imaging of the CNS was not performed in most cases.

Neuropsychiatric symptoms were not associated with the presence of headaches, indicating that other neurological manifestations of SLE are not accompanied by migraine-type headaches.

It should be noted that the SLICC criteria do not include the presence of isolated headaches as a diagnostic criterion for SLE, and only severe and persistent headaches unresponsive to narcotics are included in the SLEDAI scoring system, 16 which is an important marker of disease activity. Headaches are rarely scored on the SLEDAI scale, which may lead to an underestimation of their association with disease activity. 16 In the current study, we did not find any association between the presence of headaches and SLEDAI scores at the time of evaluation.

The present was the first study to associate worse patient perception of their health and activities, as well as of their feelings, with the presence of headaches, indicating that headaches may impact the quality of life of jSLE patients.

The limitations to the study include the small number of patients and the absence of CNS imaging for the differential diagnosis of headaches. Its strengths include its cross-sectional design with a comprehensive clinical evaluation of headaches by experienced professionals. This reinforces the importance of thorough history-taking and physical examination in investigating SLE-related headaches, especially when imaging is not easily accessible.

In conclusion, headaches were more prevalent in female jSLE patients, and most patients experienced migraines as the primary cause of this symptom. Patients with headaches presented lower scores on quality-of-life questionnaires, indicating a negative impact on their perception of health and feelings. These findings underscore the importance of a comprehensive approach to managing headaches in patients with jSLE, aimed at improving quality of life.
